# The Influence of Occupational Categories on Overall and Domain-Specific Physical Activity and the Association with Chronic Diseases. An Analysis Using the Austrian Health Interview Survey

**DOI:** 10.3390/ijerph18042148

**Published:** 2021-02-22

**Authors:** Thomas Ernst Dorner, Christian Lackinger, Sandra Haider, Igor Grabovac, Katharina Viktoria Stein

**Affiliations:** 1 Department of Social and Preventive Medicine, Center for Public Health, Medical University of Vienna, 1090 Vienna, Austria or thomas.dorner@bvaeb.at (T.E.D.); sandra.a.haider@meduniwien.ac.at (S.H.); igor.grabovac@meduniwien.ac.at (I.G.); 2 Social Insurance Fund for Public Service, Railway and Mining Industries, 1080 Vienna, Austria; christian.lackinger@bvaeb.at; 3 Karl-Landsteiner Institute for Health Promotion Research, 3454 Sitzenberg-Reidling, Austria

**Keywords:** physical exercise, physical training, exercise domains, gainful employment, profession, chronic diseases, EHIS-PAQ, interview survey

## Abstract

*Background*: The performance of physical activity (PA) in different domains varies between different occupational groups and they contribute differently to the prevention and management of chronic diseases. This study aimed to give a fuller picture of the potential influence occupational categories have on the different domains of PA among the Austrian population of working age. *Methods*: A total of 8251 gainfully employed persons in 9 major and 39 sub-major occupational groups from the Austrian Health Interview Survey 2014 were analyzed. PA was measured with the Physical Activity Questionnaire of the European Health Interview Survey (EHIS-PAQ) and the prevalence of 17 chronic diseases was obtained. *Results*: A total of 48.2% were mostly active when working, 18.4% reported transport-related PA in the upper quintile, 50.4% performed at least 150 min per week of moderate PA or cycling, 32.7% performed muscle-strengthening PA at least twice a week, and 76.3% were either mostly physically active when working or complied with the aerobic PA guidelines. As a general rule, people in physically active occupational groups tended to perform less PA in their leisure time and vice versa. Occupational groups with especially low amount of PA were Information Technology workers, directors, and secretarial staff. People with a chronic disease tended to perform less PA, but there was an interaction between occupation and chronic disease on PA. *Conclusions*: Domain-specific programs to promote PA should be developed for various occupational categories.

## 1. Introduction

Physical activity (PA) is one of the cornerstones for health and wellbeing, prevention and management of diseases, as well as a protective factor against mortality [1,2,3]. It is generally stipulated that the sedentary lifestyle, brought about by changing working conditions and leisure time activities, has contributed to an increase in non-communicable diseases [4]. However, while the scientific evidence on the benefits of physical activity in general is substantial, there is little exploration of the different domains of physical activity, namely, transportation (transport-related physical activity, TRPA), occupation (work-related physical activity, WRPA), and leisure-time. Aerobic or muscle-strengthening PA with at least moderate intensity is usually labelled as health-enhancing physical activity, (HEPA) [5] and, additionally, total physical activity (TPA) is used. While there are some overlaps in these domains (e.g., cycling is recognized in both TRPA and HEPA), they are well established categories to capture all aspects of physical activity [6].

It is commonly assumed that there exist significant divergence in the amount of physical activity in different occupational groups. Unsurprisingly, non-manual occupational groups displayed more activity overall and during leisure time, with lower levels of WRPA [7]. As early as the mid-20th century studies were published demonstrating more physical activity among the walking bus conductors in London’s double-decker busses compared to the sedentary bus drivers, which resulted in an apparent protection against cardio-vascular diseases. Additionally, physically active postmen seemed to be protected against cardio-vascular diseases, compared to less active government employees [8]. Since then, many studies have been published demonstrating differences in PA in different occupational groups with various methods for operationalizing the amount of PA, like questionnaires (self-reported data) or device-based measurements [9,10,11,12,13,14]. A set of studies using such devices have found that people working manually have more TPA and WRPA, but there were seemingly no differences according to occupation when it came to HEPA [15,16,17,18,19]. However, in most of these studies the focus lay on aerobic PA and they did not evaluate muscle-strengthening PA (MSPA). Furthermore, most studies concentrated on only a few occupational groups, and no differentiation was made between WRPA, TRPA, and HEPA.

The association between PA and chronic diseases is complex. On the one hand, lack of PA can advance the development of chronic diseases, which is expressed in lower levels of PA in persons with chronic diseases in cross sectional studies. On the other hand, PA can contribute to the therapy of chronic diseases, which would lead to higher levels of PA in persons with chronic diseases in cross sectional studies. The association between PA and chronic diseases seems to be influenced significantly by age [20]. Another important relationship, which is known as ‘physical activity paradox’, can be observed in the seemingly negative impact that work-related physical activity has on health outcomes [21,22,23,24].

In order to preserve health and gain substantial health benefits a minimum of 150 min of aerobic moderate-intensity physical activity or ≥75 min of vigorous-intensity physical activity, or an equal combination of both, is required. Additionally, muscle-strengthening physical activity (MSPA) is recommended in national and international guidelines [4,25,26]. However, a large proportion of adults do not meet these guidelines, and there are major differences between subpopulations. The physical activity status in Austria has been shown to vary according to the presence of chronic diseases [20], and socio-demographic data such as age [20,27], sex, country of birth, educational level, and relationship status [27].

This study aims to give a fuller picture of the potential influence occupational categories have on the different domains of PA among the Austrian population of working age. Another important aim was to analyze the association between chronic diseases and PA, and to ascertain potential interaction effects between occupation and chronic diseases on PA domains. Additional influencing factors like sex, educational status, and country of birth were also considered.

## 2. Materials and Methods

### 2.1. Dataset

The Health Interview Survey (AT-HIS) of 2014 formed the database of this analysis. The AT-HIS is carried out every five years in Austria, in subjects aged 15 years and older, and executed by Statistics Austria on behalf of the Austrian government. The basis for this survey is the European Health Interview Survey (E-HIS), which is regularly undertaken by the Member States of the European Union (EU) [28,29], and is adapted for the Austrian context by a national expert panel [30,31]. For the AT-HIS, the sample is stratified into 32 NUTS-3 region (nomenclature des unités territoriales statistiques), with 462 persons in each region (and 560 persons for the three regions in Vienna). The possibly distorting effect of geographic stratification is mitigated by weighing the data using the geographic region, age, sex, family situation, migration background, and education level as weighting factors. Missing values were almost negligible and have been addressed by imputation of the non-responses, based on sex, age, education, and place of residence.

The survey was carried out from October 2013 to June 2015 via computer-assisted telephone interviewing (CATI). In addition, paper questionnaires were sent out for specific topics, including the questions about physical activity, which had to be returned through regular post. The survey started with a gross sample size of 38,768 persons from the central population register. Of these, 21,343 people declined participation; another 1594 who initially declared their willingness to participate could no longer be reached, or refused the telephone interview; 25 persons terminated the interview before completion; and 35 persons were excluded due to unsatisfactory data quality. This resulted in a net sample of 15,771 persons who were included in the survey, yielding a response rate of 40.7%. The response rate for the paper-based questionnaire was 93% [30].

For the study at hand, only data for persons being gainfully employed were used in the analysis (in total 8251 persons). Excluded were unemployed persons, retired subjects, housewives and househusbands, students, people in disability pension, people in military or civil service, and subjects on parental leave.

### 2.2. Physical Activity

For evaluating the amount of PA carried out by respondents, the 8-item PA Questionnaire of the EHIS (EHIS-PAQ) was used [6]. This tool was adapted from the Global Physical Activity Questionnaire (GPAQ) [32], and examines physical activity during a typical week while at work, using different means of transportation, or performing aerobic leisure-time or muscle-strengthening activities. Only activities lasting for more than 10 min were included, according to the recommendations for health-enhancing PA [25,33]. Based on these items, 5 different binary PA indicators were formed: (1) work-related PA (WRPA) measures the percentage of subjects being mostly physically active when working; (2) transport-related PA (TRPA) summarizes the minutes per week spent with walking and transport-related cycling. To arrive at the indicator metabolic equivalent (MET)-minutes per week were calculated by multiplying the minutes per week with 3.3 METs for walking and with 6 METs for cycling, respectively, as specified in the EHIS-PAQ. The upper quintile of the MET-minutes per week was used to dichotomize this indicator; (3) compliance with the recommendations for health-enhancing PA (HEPA) was computed by summing up the minutes per week spent with cycling and with sports, fitness or recreational (leisure) physical activities. As international and national guidelines for HEPA recommend at least 150 min moderate intensity PA per week [25,33], the indicator was dichotomized as fulfilling this criterion or not; (4) the indicator for muscle-strengthening PA (MSPA) was fulfilled when performing muscle-strengthening activities (such as strength training or strengthening exercises with weights, with gymnastic bands, with the own body weight, or squats, push-ups or sit-ups) at least twice a week [6]. This is also in line with international and national recommendations [25,33]; (5) the indicator total PA (TPA) was defined by either achieving the guidelines for HEPA, or by having a job, which demanded a lot of physical activity. The EHIS-PAQ has shown good reliability and validity [34,35].

### 2.3. Occupational Categories

The professional groups were categorized according to the International Standard Classification of Occupations (ISCO-08) [36], Austrian Version (Ö-ISCO) [37]. This classification describes 10 major and 43 sub-major groups. A total of 9 major and 39 sub-major groups were represented in our sample. It needs to be acknowledged, however, that the categories used in the AT-HIS do not exactly correspond to either the German or the English version, so the authors sometimes had to translate themselves to find the best match.

### 2.4. Chronic Diseases

Participants were asked whether or not they had any chronic diseases, specified in a list of 17 chronic diseases. The diseases were bronchial asthma, chronic obstructive pulmonary disease, heart attack or chronic condition after heart attack, coronary heart diseases or angina pectoris, hypertension, stroke or chronic condition after stroke, osteoarthritis, diabetes mellitus, chronic back pain, chronic neck pain, allergy (coryza, food, dermatitis), hepatic cirrhosis, urinary incontinence, kidney disease or renal insufficiency, depression, chronic headache, and gastric or duodenal ulcers. These diseases represent the most common chronic diseases among the Austrian population and were selected by the national expert panel advising Statistics Austria on the content of the AT-HIS tool. Participants could select all that applied.

### 2.5. Sociodemographic Data

Participants’ sex (the AT-HIS only distinguishes between male and female) and age were recorded. The relationship status was represented as either ‘in a relationship’ (including being married) or ‘not in a relationship’. Education was defined in three levels: primary education (compulsory education up to age 15); secondary education (apprenticeship and vocational schools, professional/commercial schools, and high school); and tertiary education (university). Furthermore, country of birth was documented with three categories: Austria, EU states (comprising the 27 European member states for the year 2014, except Austria), and non-EU.

### 2.6. Statistical Analyses

For the statistical analyses IBM SPSS 24 (IBM, Armonk, NY, USA) was used. All the analyses were carried out with the weighted data. Bivariate analyses were performed by means of cross-tabulations, and group differences were assessed with Pearson’s Chi-squared tests. Binary logistic regression models were run with the respective PA domains as the dependent variables. Independent variables were occupation, having a chronic disease, age, sex, being in a relationship, educational level, and country of birth. All variables were used as categorical variables, except age which was used as a continuous variable. All variables were mutually adjusted. The estimates of the logistic regression models are presented as odds ratio (OR) and 95% confidence interval (95% CI). To test for the interaction between occupation and chronic disease on the different PA domains, binary logistic regression analyses were conducted, where all variables mentioned above and the product of occupation and chronic diseases were added to the models. If a significant interaction was found, the proportion of people with PA in the different domains was indicated with and without chronic diseases, stratified by occupation.

### 2.7. Ethical Considerations

The secondary analysis of the AT-HIS database was approved by the Ethics Committee of the Medical University Vienna: (EK # 2211/2015).

## 3. Results

### 3.1. Socio-Demographic Characteristics and Occupational Categories

Socio-demographic and health-related characteristics of the total sample and the different occupations are shown in Table 1. Most people were between the ages of 30 to 49. There were fewer women than men. The majority was in a relationship. More than half of the people had secondary education as the highest educational level. The vast majority was born in Austria. About one third had a chronic disease, with allergies, chronic low back pain, and chronic neck pain being the most common diseases.

There were major differences in socio-demographic and health-related characteristics in the different occupations. The most remarkable differences were the older age in managers and agriculture and forestry workers, the young age in blue-collar workers, office workers, and service workers, and the low female proportion in blue-collar workers, plant and machine operators and assemblers, and managers. Agriculture and forestry workers, and managers were most often in a relationship, while service workers and office workers were least often in a relationship. The highest proportion of people with tertiary education was found in academic laborers, and the highest proportion of people with only primary education in unskilled laborers. The highest proportion of people born outside of Austria was found in unskilled workers, service workers, and engine workers.

There was no significant difference in the proportion of people with any chronic disease in the different groups of occupation; however, looking at the details revealed interesting differences nonetheless. Managers suffered significantly more often from hypertension and heart attack, whereas academic laborers, skilled laborers, and office workers were more often affected by allergies. Service workers experienced chronic neck pain, chronic head ache, and depression more often than other occupational groups. Agriculture and forestry workers were significantly often affected by hypertension, chronic low back and neck pain. Similarly, chronic low back pain was a significant cause of ill health in blue-collar workers. This was specifically true in plant and machine operators and assemblers, who also indicated high percentages of hypertension and osteoarthritis. Unskilled laborers were the group with the highest prevalence of chronic diseases, with common diagnoses including chronic low back and neck pain, osteoarthritis, chronic headache, depression, gastric or duodenal ulcers, and a history of stroke.

### 3.2. Physical Activity

Regarding physical activity, almost half of the sample was mostly active when working (WRPA), 18.4% reported transport-related PA (TRPA) in the upper quintile of the population, about half of the sample performed at least 150 min per week of moderate PA or cycling and thus complied with the aerobic HEPA guidelines, about a third performed muscle-strengthening PA at least twice a week, and about three quarters fulfilled the requirements for TPA (were either mostly physically active when working or complied with the aerobic HEPA guidelines).

Physical activity in different occupational groups are depicted in Table 2. Managers were among the occupational groups with least physical activity, in all PA dimensions, except HEPA which was around the average. Against this general rule, among managers, executives in hotels and restaurants were very often physically active when working, executives in the commercial area fulfilled often the minimal criteria for HEPA, and directors, board members, senior administrators, and members of legislative bodies were among the occupational groups that performed most often the recommended MSPA. Academic laborers were those with least WRPA (exception: academic health professionals), but with most HEPA and MSPA. Within academic laborers, specialists in information and communication technology had especially low levels of physical activity in all domains and were the occupation group with least WRPA. Business economists and academic health professionals, however, had a remarkable high proportion of persons performing MSPA. Skilled laborers had lower levels of WRPA, TRPA, and TPA, compared to the average, but higher levels of HEPA and MSPA. Within this group, non-academic legal, social care, cultural, and related professionals had high levels of PA in all domains, especially in HEPA and MSPA. Other exceptions were assistant professions in health care, who had a high proportion of people being physically active when working. Information and communication technicians, however, were the occupational group with the lowest levels of PA in all domains. Office workers were characterized by a low amount of WRPA and average amounts in HEPA and MSPA. Within this group, general clerks and secretarial staff had an especially low amount of WRPA and TRPA. Service workers had a high amount of WRPA, a low amount of HEPA, and an average amount of MSPA. Agriculture and forestry workers were characterized by a very high amount of WRPA, TRPA, and TPA, but a low amount of HEPA and MSPA. Blue-collar workers were very often physically active when working, had a low amount of HEPA, an average amount of MSPA, and a high amount of TPA. Within this group, construction workers were remarkably often physically active when working and were the occupational group with the highest amount of TRPA, and also often participated in MSPA. Engine workers and unskilled workers were characterized by being often physically active when working, and being often in the upper TPA quintile, but not often fulfilling the criteria for HEPA and MSPA. Among unskilled workers, WRPA was especially high in cleaning staff, but this group rarely performed MSPA.

### 3.3. Physical Activity and Chronic Diseases

As Table 3 shows, people with a chronic disease tended to perform less physical activity overall, and in the domains TRPA, HEPA, and MSPA. However, their WRPA was higher when compared to those with no chronic diseases. This difference was particularly pronounced regarding bronchial asthma, COPD, hypertension, osteoarthritis, chronic low back pain, diabetes mellitus, depression, and chronic headache, whereas people with allergies were less often physically active when working compared to those without allergies. Regarding active transportation, there were no significant differences in those with a specific chronic disease compared to those without. Patients with COPD, hypertension, chronic low back pain, urinary incontinence, and depression particularly hardly ever with the endurance-based recommendations for HEPA. While people diagnosed with hypertension, urinary incontinence, and depression adhered to the recommended level of muscle-strengthening activity significantly less often than people without these diseases, in people with allergies the reverse was true. Hypertension, chronic kidney diseases, and depression significantly affected the ability to reach the upper quintile for TPA, while patients with osteoarthritis and chronic headache more often reached the criteria for TPA compared to those without these conditions.

### 3.4. Multivariate Analyses

Multivariate analyses (Table 4) revealed that factors associated with a higher amount of WRPA were agriculture and forestry workers, a lower educational level, and being born outside the EU, whereas most occupations in comparison to unskilled workers, a higher age, and male sex were associated with a lower amount of WRPA. Factors associated with a higher amount of TRPA were a higher age, male sex, and being born outside Austria in the EU, whereas being a manager, having a chronic disease, and being in a relationship lowered the chances of TRPA in the top quintile. Factors associated with a higher level of HEPA were most occupations in comparison to unskilled workers; however, chronic diseases, being in a relationship, a lower education, and having a migration background were associated with a lower level of HEPA. Most professions performed more MSPA compared to unskilled workers, in addition to men and migrants from outside the EU, whereas higher age, being in a relationship, and a lower educational level were associated with lower MSPA. Factors associated with a higher chance of fulfilling the criteria for TPA were working in agriculture and forestry, secondary education (in comparison to tertiary education), and being born outside the EU, and factors lowering the chance for TPA were most professions, having a chronic disease, and being born outside Austria but within the EU.

There was significant interaction between occupational category and presence of a chronic disease in relation to WRPA (*p* = 0.023), MSPA (*p* = 0.032), and TPA (*p* = 0.019) in the respective fully adjusted models. In the stratified analysis (Table 5), people with a chronic disease reported more often being physically active when working compared to those without chronic diseases. However, looking at the different occupations, the only occupational category where this was especially pronounced and statistically significant were unskilled laborers, whereas in other occupations (managers, skilled laborers, service workers, agriculture and forestry workers, and blue-collar workers), the associations was the other way round (those with a chronic disease performed less often PA when working), but not statistically significant. In general, people with a chronic disease had significantly lower levels of MSPA. Looking at different occupations, office workers with a chronic disease performed MSPA significantly less often, while agriculture and forestry workers with a chronic disease performed MSPA significantly more often than their counterparts with no chronic disease. This also led to significantly lower levels of TPA in people with a chronic disease. In the different occupations, this association was most significant in service workers, and blue-collar workers. On the other hand, unskilled laborers with a chronic disease had a higher amount of TPA compared to people with no chronic disease.

## 4. Discussion

Our study represents the first comprehensive analysis of domain-specific PA among people of working age covering all occupational categories represented in the Austrian Health Information Survey (ATHIS), and also the relation of chronic diseases with PA in different occupational groups. Unsurprisingly, working conditions and occupational categories had a significant influence on work-related physical activity, as is exemplified by the difference in WRPA between managers and agriculture and forestry workers. The presence of chronic diseases also influenced PA in the different PA domains to varying degrees, and different occupations apparently coped differently with having a chronic condition and following PA.

Underlying these analyses is also a distinct gender bias regarding occupational categories, but this does not seem to affect the domains of physical activity as much as might be expected (which can be also seen in the multivariate models, where sex was adjusted). Service workers, office workers, and unskilled laborers are disproportionately female and all show low to average levels of health-related physical activity and MSPA. At the same time, blue-collar workers and engine workers are predominantly male categories, but they also underperform in HEPA and MSPA. This already illustrates that for people in physically demanding jobs it is difficult to cover other domains of physical activity and therefore targeted programs would be beneficial. In the Finnish Retirement and Aging Study [38], which used the same professional categories as our study and similar PA domains, gender seemed to play a more significant role, with women having a higher overall activity level than men, even after adjusting for factors like chronic diseases or living area. The sex differences were also observed between professional categories; however, the differences in activity levels between professional categories was less pronounced in men than women. The authors surmised that much of the gender-based differences were due to the heavily segregated Finnish job market, but Austria sees similarly skewed percentages. The other reason was found in the higher percentage of Finnish women actively commuting to work.

Managers are another interesting category, as the industry they represent clearly influences what type of physical activity dominates, even though they all seem to prefer HEPA. However, their overall activity level is low and TRPA is virtually non-existent, which makes it even more important for this group to realize that daily activity levels can be boosted through active modes of transport and even short intervals throughout the day.

### 4.1. Work-Related Physical Activity and Health Effects

At this point, the so-called physical activity paradox needs to be discussed, which has received wide attention in several studies [21,22,23,24]. This paradox questions the health benefits of WRPA, and many studies conclude that there is no beneficial effect of WRPA. In one meta-analysis covering over 200,000 participants, the authors even found detrimental effects and increased all-cause mortality in men in relation to occupational physical activity [39]. As most studies on physical activity levels and health benefits only consider leisure time and transport-related PA, no definitive conclusions can be drawn yet. However, it is clear that it is necessary to take a closer look at the different forms of physical activity during work. One reason for the negative effects may be found in the monotonous, repetitive, and one-sided motions of many physically demanding jobs [40]. Along with that comes the fact that many movements are not performed safely (e.g., lifting weight to cause spinal or back injuries). Unsafe, polluted, or otherwise unhealthy working environments (e.g., dust, noise, heat) may also contribute to long-term health issues and confound the outcomes for PA. Holterman et al. (2018) [40] postulated six hypotheses to explain these underlying mechanisms of the PA paradox. In an interesting study [24], comparing relative time spent on moderate-to-vigorous PA during work versus during leisure time showed an increased risk for long-term sickness absence in the former group, and a reduced risk in the latter. This emphasizes the importance of differentiating between the forms of physical activity, and where and when they are performed. In our study, a special focus needs to be put on information technology (IT) workers (academic and non-academic), who basically do not seem to move at all.

Another aspect, which Holterman et al. (2018) [40] discussed, was the low control of most manual workers over their work and working environment and the low socio-economic level of many of them. Early studies [41,42] have hypothesized that high-strain jobs (high psychological demands and low control) require longer recovery times, or low self-efficacy caused by low-demand, repetitive jobs may lead to lower physical activity. These hypotheses were confirmed by a meta-analysis [43] with 170,000 participants, which found 21–26% higher odds for physical inactivity in people with high-strain and passive jobs.

These distinctions are mirrored to some extent in the difference in educational levels for certain domains of physical activity, where HEPA and MSPA are mostly performed by people with higher educational levels. However, similar to other studies [38,44], virtually no difference between educational levels could be detected when looking at TRPA and TPA. The real surprise in our study was that WRPA was highest among people with secondary education, with almost three times the level of those with tertiary education. Educational level alone clearly does not enhance physical activity.

### 4.2. Chronic Disease and Physical Activity

The relationship between chronic diseases and PA requires an in-depth analysis. While people with chronic diseases reached the thresholds for physical activities in the domains transport, health enhancing, muscle strengthening, and in total less often, they were significantly more often physically active when working (especially in unskilled laborers). Furthermore, the association between chronic diseases and PA was differently pronounced in different occupational groups, as the interaction analysis revealed.

In the multivariate analysis, however, there was no association between WRPA and chronic disease. That means that the counterintuitive positive association between WRPA and chronic disease was not facilitated by work-related factors as such, but by socio-demographic factors which the analyses was adjusted for. For example, it is likely that unskilled laborers with chronic diseases work more often in occupations that require a high amount of work-related PA. In that case, there is not really a causal association between chronic disease and WRPA, but both factors are an expression of a common underlying factor, such as low socio-economic position.

Finally, the association between having a chronic disease and performing less PA in the domains TRPA, HEPA, and TPA, according to the multivariate regression, remains to be discussed. As always in cross-sectional studies, we cannot draw final conclusions regarding cause and consequence; however, it is plausible that persons with chronic diseases perform less PA, because the lack of PA has contributed to the development of chronic diseases. This is especially true for the chronic diseases hypertension, diabetes mellitus, or depression, where the PA differences between those who are affected versus those who are not affected are remarkably pronounced in our data. On the other hand, it is also plausible that they perform less PA, because chronic diseases negatively influence functionality and therefore hinder PA. This can be especially the case regarding chronic low back pain, and again depression, where the differences in PA between those affected and those not affected were very pronounced. In any case, people with chronic diseases should actually perform PA at least in the amount of the recommendations for HEPA and MSPA, and this is definitely largely not the case. The difference in TPA between those affected versus not affected by a chronic disease was especially pronounced in service workers and blue-collar workers, and in this domain there was also a significant interaction effect between occupations and the presence of a chronic disease on PA. Therefore, occupation must be taken into account when recommending patients with chronic diseases towards more PA.

It has to be acknowledged here that we did not analyze multi-morbidity and physical activity. The primary focus of this study was on physical activity and professional categories, and not on the influence of chronic disease on physical activity. Having said that, this would certainly be worthwhile further investigation.

### 4.3. Strengths and Limitations

The sample size is large and representative, which is a clear strength of the study. This is due to the fact that the Austrian Central Population Register was used to draw the sample, which represents the general population, and allowed for the weighting of the sample according to various socio-demographic variables. The population-based design of the study permitted the combined analysis of healthy subjects with subjects with chronic diseases. The large sample size also enabled the analysis of 39 different occupational groups and the additional use of the EHIS-PAQ delivered the different domains of PA. Both factors are rarely found in comparable surveys. However, although the total sample size was large, some subgroups were relatively small, yielding a limited power for the statistical analyses. Another potential limitation is that all the factors analyzed were self-reported. This may have led to overestimation of PA, since people may have given socially desirable answers or simply overestimated their own level of PA, as well as underestimation of the prevalence of chronic diseases.

### 4.4. Recommendations for Policy and Practice

This analysis helps inform recommendations for policymakers, employers, and employees in supporting physical activity across all domains. Domain-specific programs should be developed for various occupational categories, such as IT workers, managers, or unskilled workers addressing their needs. A general promotion of transport-related PA, advertising walking and cycling for commuting trips or at least parts of them, would support a higher TPA, and is an easy and low-resource intervention. Regarding WRPA, there is a clear need to counteract adverse effects from monotonous, repetitive, and unsafe motions and support manual workers in keeping up their overall physical activity. These measures will not only support better health outcomes, but also contribute to a healthier workforce, staying longer in the labor market, and accruing fewer sick days.

## 5. Conclusions

It is undisputed that physical activity contributes to better health outcomes, reduces the risk of chronic diseases, slows their progress, and improves general mental and physical health and wellbeing. However, when looking into the different domains of physical activity, a much more complex picture emerges, which highlights the intricate interplay of many external factors, such as socio-demographic parameters. A special focus needs to be put on work-related physical activity, as the evidence increases that the nature of it actually increases mortality rates, rather than leading to health benefits.

## Figures and Tables

**Table 1:** Socio-demographic and health-related characteristics of the gainfully employed persons in Austria in total and in different occupational groups (all values in %).

Characteristics	Total Sample	Managers	Academic Laborer	Skilled Laborer	Office Worker	Service Workers	Agriculture and Forestry Workers	Blue-Collar Workers	Engine Workers	Unskilled Laborer	*p*
Age 15–29 y	20.4	5.7	15.2	21.9	25.4	25.1	10.8	30.6	12.7	11.2	<0.001
Age 30–49 y	52.6	54.2	56.2	52.4	52.4	49.6	50.6	48.7	59.3	52.8	
Age 50+ y	27.0	40.0	28.6	25.7	22.0	25.3	38.6	20.7	28.0	36.0	
Female sex	44.9	22.7	46.9	43.0	71.0	68.3	39.8	9.6	11.8	62.6	<0.001
In relationship	72.4	81.5	74.8	72.6	69.6	65.4	82.2	71.2	74.4	78.2	<0.001
Primary education	11.9	4.6	1.5	5.4	11.7	19.4	15.6	12.3	18.1	44.5	<0.001
Secondary education	53.5	38.7	14.3	54.9	54.1	67.5	75.5	79.7	73.0	48.4	
Tertiary education	34.6	56.8	84.2	39.8	34.2	13.1	8.9	7.9	8.8	7.1	
Born in Austria	82.6	86.0	82.3	86.0	87.0	77.9	98.5	81.4	78.2	69.9	<0.001
Born in EU	10.7	9.8	13.9	9.6	8.7	13.5	1.1	8.5	9.1	13.9	
Born outside EU	6.7	4.1	3.8	4.3	4.2	8.6	0.4	10.1	12.7	16.2	
Any chronic disease	28.0	27.0	27.2	26.8	30.7	29.8	29.4	26.4	27.2	28.7	0.330
Bronchial asthma	3.3	3.7	3.0	2.7	3.3	3.7	5.2	3.4	3.6	4.0	0.547
Chronic obstructive pulmonary disease	2.2	3.0	1.9	1.7	2.0	2.5	3.7	1.9	3.4	3.1	0.107
Heart attack or chronic condition after heart attack	0.3	1.1	0.2	0.2	0.1	0.1	0.0	0.8	0.2	0.6	0.015
Coronary heart diseases or angina pectoris	0.7	1.4	0.6	0.5	1.1	0.7	0.7	1.0	0.5	0.4	0.497
Hypertension	11.9	14.4	10.2	11.2	10.1	13.1	14.1	12.0	14.7	12.1	0.033
Stroke or chronic condition after stroke	0.2	0.2	0.2	0.2	0.0	0.1	0.0	0.3	0.2	1.2	0.012
Osteoarthritis	5.2	5.5	4.7	4.0	5.1	5.6	5.6	4.7	6.3	10.0	<0.001
Chronic low back pain	19.2	15.1	14.6	17.1	16.6	21.4	27.5	21.0	25.9	28.7	<0.001
Chronic neck pain	15.9	14.2	14.6	16.3	17.0	18.1	19.3	12.1	13.6	20.4	<0.001
Diabetes mellitus	1.8	2.3	1.4	2.1	2.0	1.9	1.1	1.8	1.1	1.5	0.729
Allergy (coryza, food, dermatitis)	25.6	24.3	29.1	28.0	28.2	26.6	18.6	20.4	18.1	22.9	<0.001
Hepatic cirrhosis	0.1	0.0	0.0	0.2	0.1	0.1	1.1	0.2	0.0	0.0	0.051
Urinary incontinence	0.6	0.0	0.4	0.4	0.9	0.5	1.5	0.2	1.6	1.9	<0.001
Kidney disease or renal insufficiency	0.5	0.5	0.5	0.5	0.6	0.7	0.7	0.1	0.0	0.6	0.469
Depression	4.2	3.0	3.8	3.7	3.9	6.3	4.1	2.0	3.4	7.9	<0.001
Chronic headache	6.0	2.7	4.7	5.4	7.1	9.0	4.1	4.9	5.2	8.7	<0.001
Gastric or duodenal ulcers	1.7	1.4	1.2	1.3	1.4	2.6	2.2	1.5	2.3	3.1	0.014

**Table 2:** Proportion of people who reached the respective thresholds in the different domains of physical activity among gainfully employed persons in Austria in 39 different occupational groups.

Occupational Category and Subgroups	WRPA	TRPA	HEPA	MSPA	TPA
Total	48.2	18.4	50.4	32.7	76.3
Manager (*N* = 437)	23.3	14.0	52.7	28.8	68.6
Directors, boards of directors, senior administrators, and members of legislative bodies (*N* = 44)	4.5	13.6	55.6	42.2	59.1
Executives in the commercial area (*N* = 162)	11.1	16.0	61.3	33.7	68.5
Executives in production and special services (*N* = 152)	20.4	12.4	52.3	24.8	66.0
Executives in hotels and restaurants, in retail, and in the provision of other services (*N* = 78)	64.1	12.8	33.3	18.2	79.5
Academic laboror (*N* = 1435)	19.2	19.8	61.9	36.6	68.1
Scientists, mathematicians, and engineers (*N* = 245)	6.5	18.3	62.4	33.9	65.4
Academic Health Professions (*N* = 171)	46.8	19.2	59.3	39.5	76.2
Teachers (*N* = 428)	26.9	20.4	64.6	36.4	72.2
Business economists and comparable academic professions (*N* = 244)	9.0	18.4	61.1	41.4	63.9
Academic and comparable specialists in information and communication technology (*N* = 168)	3.6	17.2	56.8	29.2	59.5
Lawyers, social scientists, and cultural professions (*N* = 178)	19.7	25.8	63.3	38.2	67.4
Skilled laboror (*N* = 1870)	37.9	16.4	54.3	34.2	70.7
Engineering and comparable skilled workers (*N* = 468)	47.2	19.1	52.6	35.5	76.9
Assistant professions in health care (*N* = 362)	72.9	21.0	57.7	34.3	87.6
Non-academic business and commercial specialists and administrative specialists (*N* = 791)	13.5	12.8	53.1	32.1	59.5
Non-academic legal, social care, cultural, and related professionals (*N* = 159)	63.5	19.0	64.3	44.3	80.4
Information and communication technician (*N* = 159)	18.7	12.1	44.0	26.7	52.7
Office worker (*N* = 848)	20.9	15.4	53.6	33.4	64.9
General clerks and secretarial staff (*N* = 386)	14.8	11.1	50.9	30.1	59.3
Office workers with customer contact (*N* = 183)	15.3	21.4	61.0	30.7	67.6
Office workers in finance and accounting, in statistics, and in materials management (*N* = 205)	21.5	15.2	56.4	35.3	66.3
Other office workers and related professions (*N* = 75)	65.3	24.0	41.3	26.7	82.7
Service workers (*N* = 1416)	67.7	19.1	44.4	32.3	82.4
Personal service professions (*N* = 415)	72.0	17.8	42.7	35.7	83.4
Sales force (*N* = 658)	63.7	17.6	41.7	28.7	80.7
Care professions (*N* = 250)	77.6	22.8	50.0	35.2	87.6
Protection forces and security guards (*N* = 92)	48.9	25.0	56.5	35.9	76.1
Agriculture and forestry workers (*N* = 269)	92.9	24.5	31.2	22.6	95.9
Skilled workers in agriculture (*N* = 260)	92.7	25.3	31.8	23.0	95.8
Skilled workers in forestry, fishing, and hunting (*N* = 9)	100	11.1	12.5	12.5	100
Blue-collar workers (*N* = 1031)	79.0	20.3	46.2	34.8	89.0
Construction specialists and related professions (*N* = 302)	86.1	26.0	49.5	39.3	95.4
Metalworkers, mechanics, and allied professions (*N* = 354)	79.4	15.8	44.4	32.8	88.4
Precision craftsmen, printers, and handicraft professions (*N* = 43)	74.4	23.8	45.2	32.6	83.7
Electricians and electronics technicians (*N* = 156)	78.2	21.8	43.6	32.5	88.5
Food processing, woodworking and garment manufacturing, and related craft professions (*N* = 175)	68.6	17.6	46.3	33.7	80.7
Engine workers (*N* = 440)	64.5	18.8	47.6	28.1	82.3
Operators of stationary systems and machines (*N* = 114)	87.7	18.6	56.1	21.1	93.0
Assembly workers (*N* = 34)	85.3	11.8	44.1	26.5	88.2
Vehicle drivers and operators of mobile systems (*N* = 293)	52.9	19.5	44.7	31.1	77.1
Unskilled labouror (*N* = 482)	82.8	20.9	32.4	22.2	87.3
Cleaning staff and auxiliary workers (*N* = 293)	93.4	20.4	34.7	15.8	95.4
Unskilled laborers in agriculture, forestry, and fishing (*N* = 3)	33.3	0	33.3	0	33.3
Unskilled laborers in mining, construction, manufacturing, and transportation (*N* = 189)	79.9	21.7	34.4	30.7	86.2
Food preparation assistants (*N* = 40)	77.5	22.5	17.5	7.5	82.5
Street vendors and street service workers (*N* = 3)	66.7	0	100	66.7	100
Waste disposal workers and other auxiliary workers (*N* = 51)	60.8	11.8	23.5	25.5	67.3
*p*	<0.001	<0.001	<0.001	<0.001	<0.001

WRPA = work-related physical activity (mostly physically active when working); TRPA = transport-related physical activity (upper quintile of metabolic equivalent (MET)-minutes/week spent with walking and cycling); HEPA = health-enhancing physical activity (at least 150 min per week moderate PA or cycling); MSPA = muscle-strengthening physical activity (at least twice per week muscle-strengthening activity); TPA = total physical activity (mostly physically active when working OR complying with HEPA guidelines).

**Table 3:** Proportion of people who reached the respective thresholds in the different domains of physical activity in gainfully employed persons in Austria with and without specific chronic diseases.

Chronic Disease	Nr of People Who Have It (Yes) or Not (No)	WRPA	TRPA	HEPA	MSPA	TPA
Total		48.2	18.4	50.4	32.7	76.3
Any chronic disease	Yes (*N* = 2306)	49.1	16.9	47.1	30.7	74.6
	No	47.9 *	18.9 *	51.7 **	33.4 *	76.9 *
Bronchial asthma	Yes (*N* = 275)	57.1	20.7	46.9	32.7	80.4
	No	47.9 *	18.3	50.6	32.7	76.1
Chronic obstructive pulmonary disease	Yes (*N* = 183)	62.1	17.5	42.1	27.3	82.0
	No	47.9 **	18.4	50.6 *	32.8	76.1
Heart attack or chronic condition after heart attack	Yes (*N* = 28)	50.0	28.6	44.4	37.0	75.0
	No	48.2	18.4	50.5	32.7	76.3
Coronary heart diseases or angina pectoris	Yes (*N* = 57)	42.1	24.6	50.9	29.8	73.7
	No	48.3	18.3	50.4	32.7	76.3
Hypertension	Yes (*N* = 979)	51.3	17.6	44.5	26.3	73.2
	No	47.8 **	18.5	51.2 **	33.5 **	76.7 *
Stroke or chronic condition after stroke	Yes (*N* = 16)	64.7	31.3	41.2	17.6	81.3
	No	48.2	18.4	50.4	32.7	76.3
Osteoarthritis	Yes (*N* = 429)	57.5	20.7	51.6	34.5	83.0
	No	47.7 **	18.3	50.4	32.6	75.9 *
Chronic low back pain	Yes (*N* = 1586)	56.5	18.3	45.9	31.8	77.3
	No	46.2 **	18.4	51.5 **	32.9	76.0
Chronic neck pain	Yes (*N* = 1315)	50.6	18.5	49.0	31.3	76.4
	No	47.8	18.4	50.7	32.9	76.3
Diabetes mellitus	Yes (*N* = 148)	50.7	19.6	42.6	25.7	69.6
	No	48.2 *	18.4	50.6	32.8	76.4
Allergy (coryza, food, dermatitis)	Yes (*N* = 2115)	46.6	17.3	52.1	36.2	76.5
	No	48.8 *	18.8	49.9	31.4 **	76.2
Hepatic cirrhosis	Yes (*N* = 12)	58.3	41.7	50.0	25.0	91.7
	No	48.2	18.3	50.4	32.7	76.3
Urinary incontinence	Yes (*N* = 51)	62.0	7.8	35.3	13.7	79.5
	No	48.1	18.5	50.5 *	32.8 *	76.3
Kidney disease or renal insufficiency	Yes (*N* = 29)	44.7	17.9	66.7	41.0	71.7
	No	48.2	18.4	50.4 *	32.6	76.5 *
Depression	Yes (*N* = 346)	51.2	16.5	39.0	20.5	71.7
	No	48.1 *	18.5	50.9 **	33.2 **	76.5 *
Chronic headache	Yes (*N* = 496)	56.6	15.9	47.8	34.7	80.8
	No	47.7 **	18.5	50.6	32.5	76.0 *
Gastric or duodenal ulcers	Yes (*N* = 142)	58.0	16.2	44.8	35.2	81.1
	No	48.1	18.4	50.5	32.6	76.2

* *p* < 0.05, ** *p* < 0.001, WRPA = work-related physical activity (mostly physically active when working); TRPA = transport-related physical activity (upper quintile of MET-minutes/week spent with walking and cycling); HEPA = health-enhancing physical activity (at least 150 min per week moderate PA or cycling); MSPA = muscle-strengthening physical activity (at least twice per week muscle-strengthening activity); TPA = total physical activity (mostly physically active when working OR complying with HEPA guidelines).

**Table 4:** Factors associated with physical activity in different domains: Results of multivariate binary logistic regression model, all factors mutually adjusted for each other.

Occupational Category	WRPA		TRPA		HEPA		MSPA		TPA	
	OR	95% CI	OR	95% CI	OR	95% CI	OR	95% CI	OR	95% CI
Manager	0.10	0.07–0.14	0.59	0.41–0.85	1.67	1.26–2.21	1.16	0.85–1.59	0.32	0.24–0.46
Academic laborer	0.10	0.07–0.13	0.97	0.73–1.30	2.26	1.78–2.88	1.59	1.22–2.07	0.33	0.24–0.45
Skilled laborer	0.16	0.13–0.21	0.76	0.58–1.00	1.88	1.50–2.35	1.52	1.18–1.94	0.34	0.25–0.46
Office worker	0.06	0.05–0.08	0.78	0.58–1.05	1.93	1.51–2.46	1.58	1.21–2.06	0.28	0.19–0.35
Service workers	0.43	0.33–0.57	0.98	0.75–1.27	1.47	1.18–1.84	1.54	1.20–1.97	0.64	0.47–0.87
Agriculture and forestry workers	2.90	1.72–4.91	1.27	0.88–1.83	0.79	0.57–1.10	0.97	0.68–1.40	3.05	1.58–5.88
Blue-collar workers	0.79	0.59–1.06	0.91	0.68–1.21	1.52	1.20–1.93	1.45	1.11–1.88	1.05	0.73–1.48
Engine workers	0.39	0.28–0.54	0.80	0.57–1.12	1.66	1.26–2.18	1.13	0.83–1.53	0.62	0.43–0.90
Unskilled laborer	1		1		1		1		1	
Chronic disease	1.08	0.97–1.21	0.87	0.77–0.99	0.84	0.76–0.93	0.96	0.86–1.07	0.89	0.79–0.99
No chronic disease	1		1		1		1		1	
Age (5 years)	0.95	0.93–0.97	1.03	1.01–1.06	1.01	0.99–1.03	0.95	0.93–0.97	0.99	0.96–1.01
Male sex	0.85	0.76–0.96	1.38	1.22–1.56	1.07	0.97–1.18	1.34	1.21–1.49	1.01	0.90–1.13
Female sex	1		1		1		1		1	
In relationship	1.06	0.94–1.19	0.79	0.70–0.90	0.80	0.76–0.93	0.72	0.65–0.80	0.91	0.81–1.03
Not in relationship	1		1		1		1		1	
Primary education	2.37	1.96–2.86	1.05	0.85–1.30	0.56	0.47–0.66	0.74	0.62–0.89	1.00	0.82–1.22
Secondary education	2.73	2.39–3.11	1.02	0.87–1.18	0.74	0.66–0.83	0.81	0.72–0.91	1.22	1.07–1.40
Tertiary education	1		1		1		1		1	
Born outside Austria in EU	0.95	0.80–1.12	1.22	1.02–1.46	0.82	0.71–0.95	0.94	0.81–1.10	0.70	0.60–0.82
Born outside Austria outside EU	1.53	1.24–1.89	1.02	0.81–1.29	0.62	0.51–0.74	1.22	1.01–1.48	1.17	0.93–1.47
Born in Austria	1		1		1		1		1	
R^2^	0.342		0.015		0.054		0.033		0.081	

WRPA = work-related physical activity (mostly physically active when working); TRPA = transport-related physical activity (upper quintile of MET-minutes/week spent with walking and cycling); HEPA = health-enhancing physical activity (at least 150 min per week moderate PA or cycling); MSPA = muscle-strengthening physical activity (at least twice per week muscle-strengthening activity); TPA = total physical activity (mostly physically active when working OR complying with HEPA guidelines).

**Table 5:** Proportion of people who reached the respective thresholds in the different domains of physical activity in gainfully employed persons in Austria, stratified by the presence or absence of any chronic disease.

Occupational Category	WRPA			MSPA			TPA		
	With a Chronic Disease	Without a Chronic Disease	*p*	With a Chronic Disease	Without a Chronic Disease	*p*	With a Chronic Disease	Without a Chronic Disease	*p*
All	49.1	47.9	0.007	30.7	33.4	0.018	74.6	76.9	0.024
Manager	22.9	23.5	0.890	32.2	27.3	0.311	71.2	67.7	0.487
Academic laborer	19.7	19.0	0.739	38.4	35.9	0.392	65.7	69.0	0.242
Skilled laborer	38.6	37.7	0.720	31.2	35.3	0.100	70.4	70.9	0.831
Office worker	23.8	19.6	0.157	28.1	35.7	0.031	60.4	66.8	0.070
Service workers	66.4	68.2	0.494	31.0	32.9	0.495	78.0	84.2	0.005
Agriculture and forestry workers	89.9	93.7	0.278	30.4	19.4	0.049	93.7	96.8	0.232
Blue-collar workers	76.8	79.8	0.296	30.1	36.5	0.059	85.7	90.1	0.046
Engine workers	69.2	62.6	0.201	22.5	30.2	0.109	82.5	82.0	0.900
Unskilled laborer	92.8	78.7	<0.001	17.4	23.9	0.119	94.2	84.5	0.004

Only WRPA, MSPA, and TPA showed a significant interaction between occupation and chronic diseases towards physical activity in the respective domains, in the fully adjusted models, and are therefore represented in the table. WRPA = work-related physical activity (mostly physically active when working); MSPA = muscle-strengthening physical activity (at least twice per week muscle-strengthening activity); TPA = total physical activity (mostly physically active when working OR complying with HEPA guidelines).

## Data Availability

The AT-HIS survey data can be obtained from Statistik Austria for research and educational purposes. For more information, please visit: http://www.statistik.at/web_de/services/mikrodaten_fuer_forschung_und_lehre/datenangebot/standardisierte_datensaetze_sds/index.html (accessed on 23 December 2020).
